# Supplementary material for: Rab6A as a Pan-Astrocytic Marker in Mouse and Human Brain, and Comparison with Other Glial Markers (GFAP, GS, Aldh1L1, SOX9)
Source: Cells. 2021 Jan 5;10(1):72. doi: 10.3390/cells10010072 (PMC7824777; doi:10.3390/cells10010072)
Supplement: Supplementary file 1 [file cells-10-00072-s001.pdf]

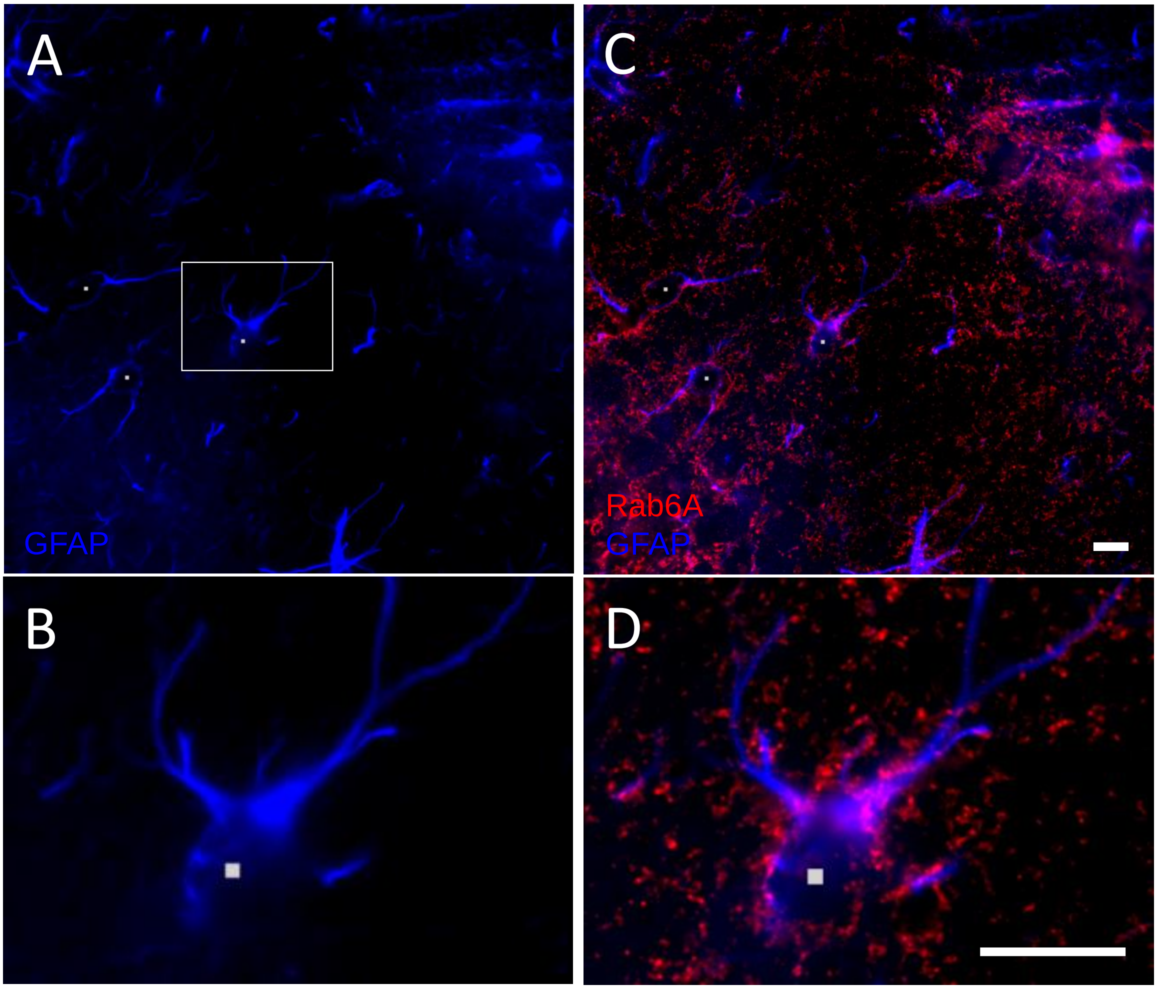

Figure S1. Procedure for assessing cellular colocalization without morphological bias. (**a**) In full frames (100x) and with only the reference channel visible (here: GFAP), cells were preselected and marked (white squares). Criteria were obvious soma with hollow nucleus and stem processes in the section plane (**b**, frame magnied from **a**). Intensely labelled processes (top right in **a**) or debatable structures, maybe vessels (top left corner) were not considered. (**c**) After overlaying the channel in question (here Rab6A), only the cells marked were assessed for double labelling, preferentially by zooming in (**d**), and included in the overall count. Cells are regarded as double labelled when displaying intense perinuclear Rab6A label. Hippocampus, stratum oriens. Scales: 10 µm.

Fig. S1

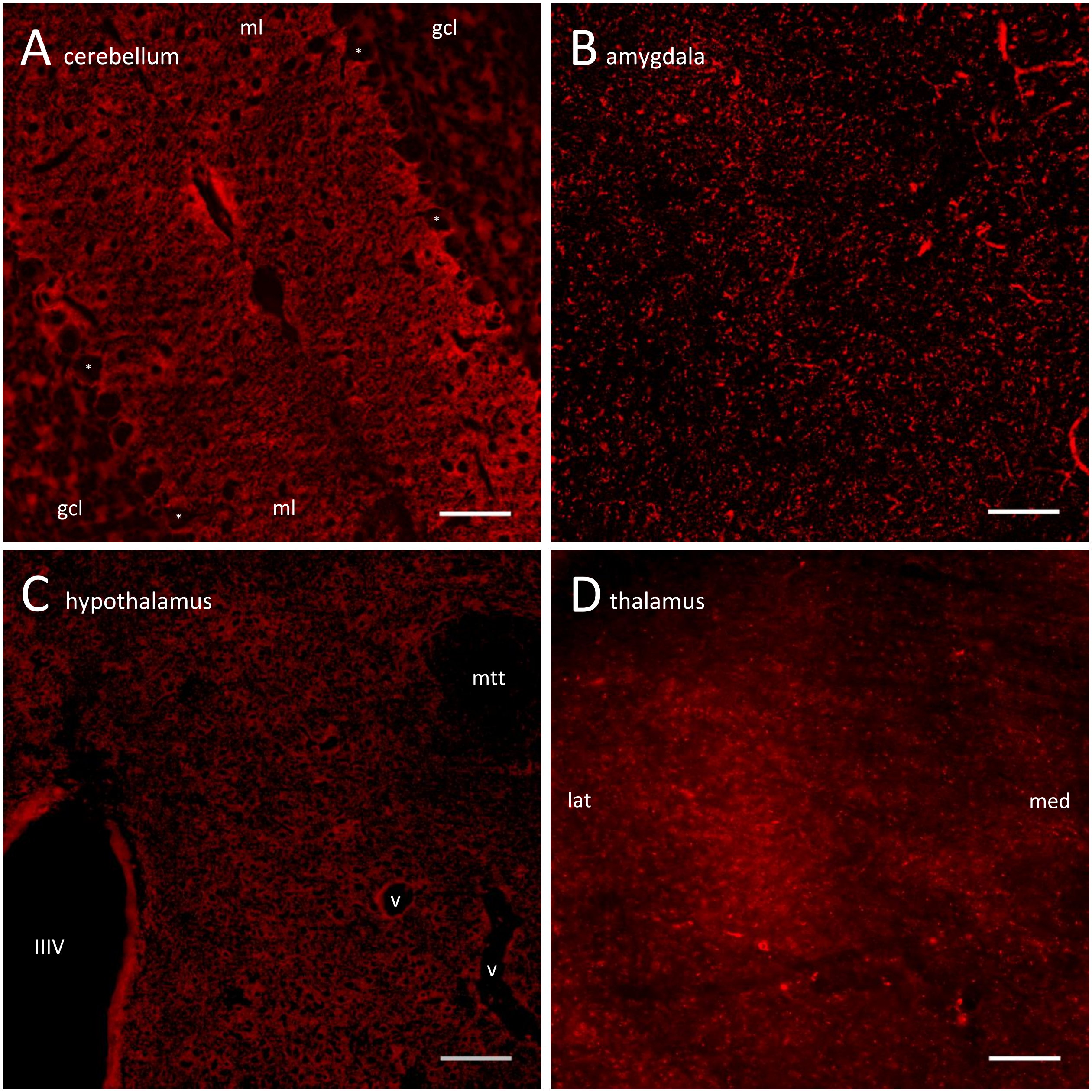

Figure S2. Rab6A staining is ubiquitous in all brain regions, as exemplified in four other regions. (**a**) Cerebellar cortex - with the pial surface of two contacting folia from upper left to lower right. Intense staining in the molecular layers (ml) and and granule cell layers (gcl). Note that glial processes negatively outline the cross-sectioned `empty' Purkinje cells, in some cases together with their proximal dendrites (asterisks). Many profiles of neuronal somata and dendrites within the molecular layer can be recognized. The shape of somata and dendrites, which are randomly organized in the amygdala (**b**) and the hypothalamus (**c**) are similarly outlined, even at lower magnification. (**d**) Rab6A staining is dense in the thalamus, spared striation (upper right) are white matter tracts of thalamocortical connections.

lat, laterat, towards cortex; med, medial, towards midline; mtt, mamillothalamic tract; IIIV, third ventricle; v, blood vessels. Scales: 50 µm (a), 100 µm (b - d).

Fig. S2

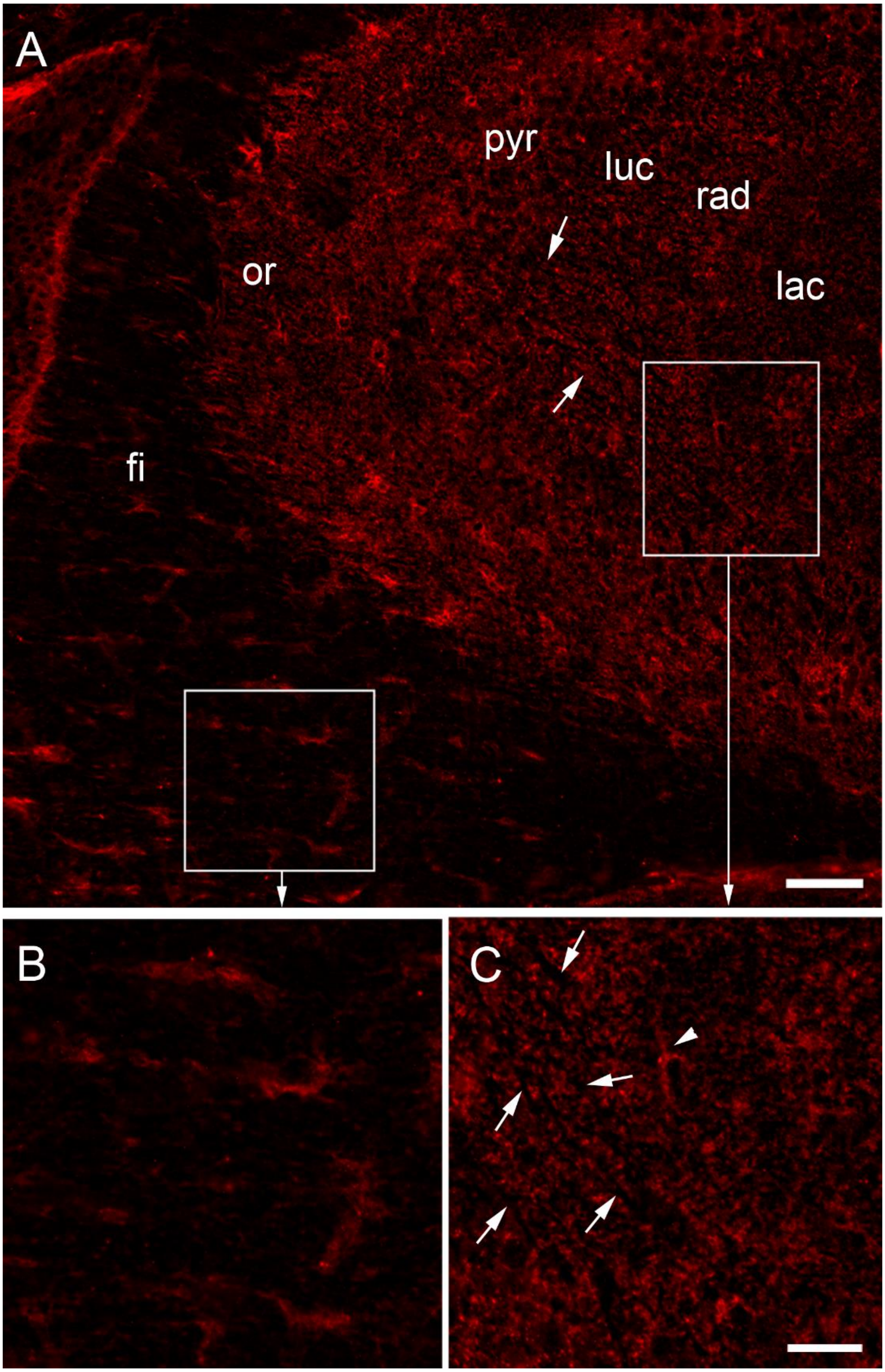

Figure S3. Different patterns in Rab6A^{+} staining are typical of astrocytes. Rab6A staining in mouse hippocampus, fimbria/ CA3 transition. (**a**) At medium magnification (20x), white matter (fimbria, lower left) displays obviously less dense staining than grey matter (upper right). The ventricular ependyma overlying the fimbria displays the honeycomb-like labelling pattern (A, top left margin). (**b**) Magnification of fimbria with interfascicular astrocytes and cut-off processes between unstained, nearly horizontal fibres. Within neuropil of CA3 (**c**, magnified from a), astrocytic cells are hardly obvious (arrowhead), however neuronal dendrites in cross and longitudinal section (arrows) are negativel outlined by the profuse granular astrocytic staining.

fi, fimbria; lac, stratum lacunosum-moleculare; luc, stratum lucidum; or, stratum oriens; pyr, stratum pyramidale; rad, stratum radiatum. Scales: 50 µm (a), 3 µm (in c, for b and c).

Fig. S3

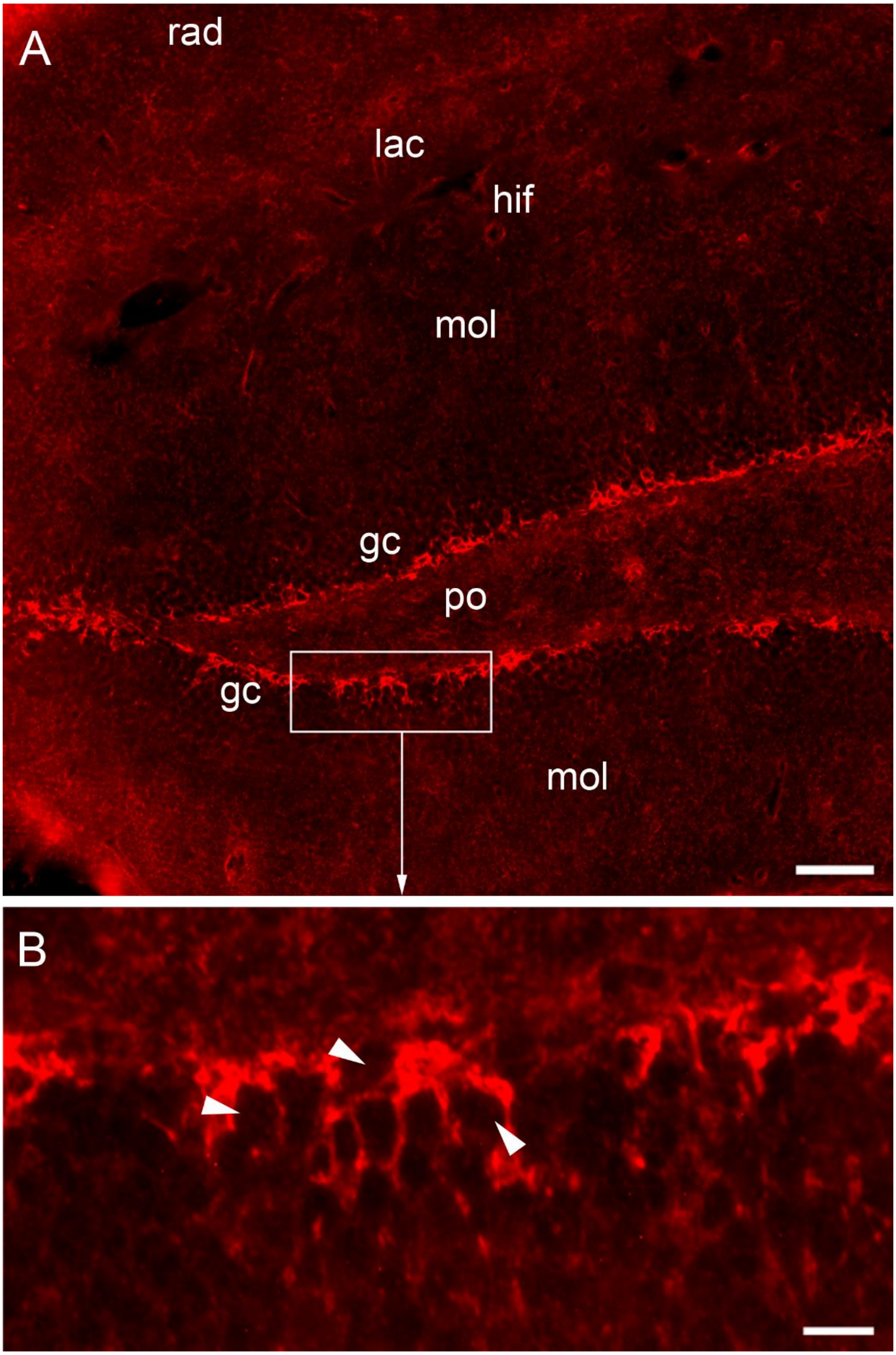

Figure S4. Rab6A⁺ structures in the fascia dentata. (**a**) Low magnification reveals differences in staining intensity. The subgranular zone and the granule cell layer (magnified in **b**) display highest staining, with the granule cells negatively outlined (arrowheads in b) and obvious subgranular cell bodies. Astrocyte territories are faintly indicated by the fluffy pattern in molecular layer.

gc, granule cell layer; hif, hippocampal fissure; lac, stratum lacunosum-moleculare; mol, molecular cell layer; po, polymorph layer; rad, stratum radiatum of CA1. Scales: 50 µm (a), 10 µm (b).

Fig. S4

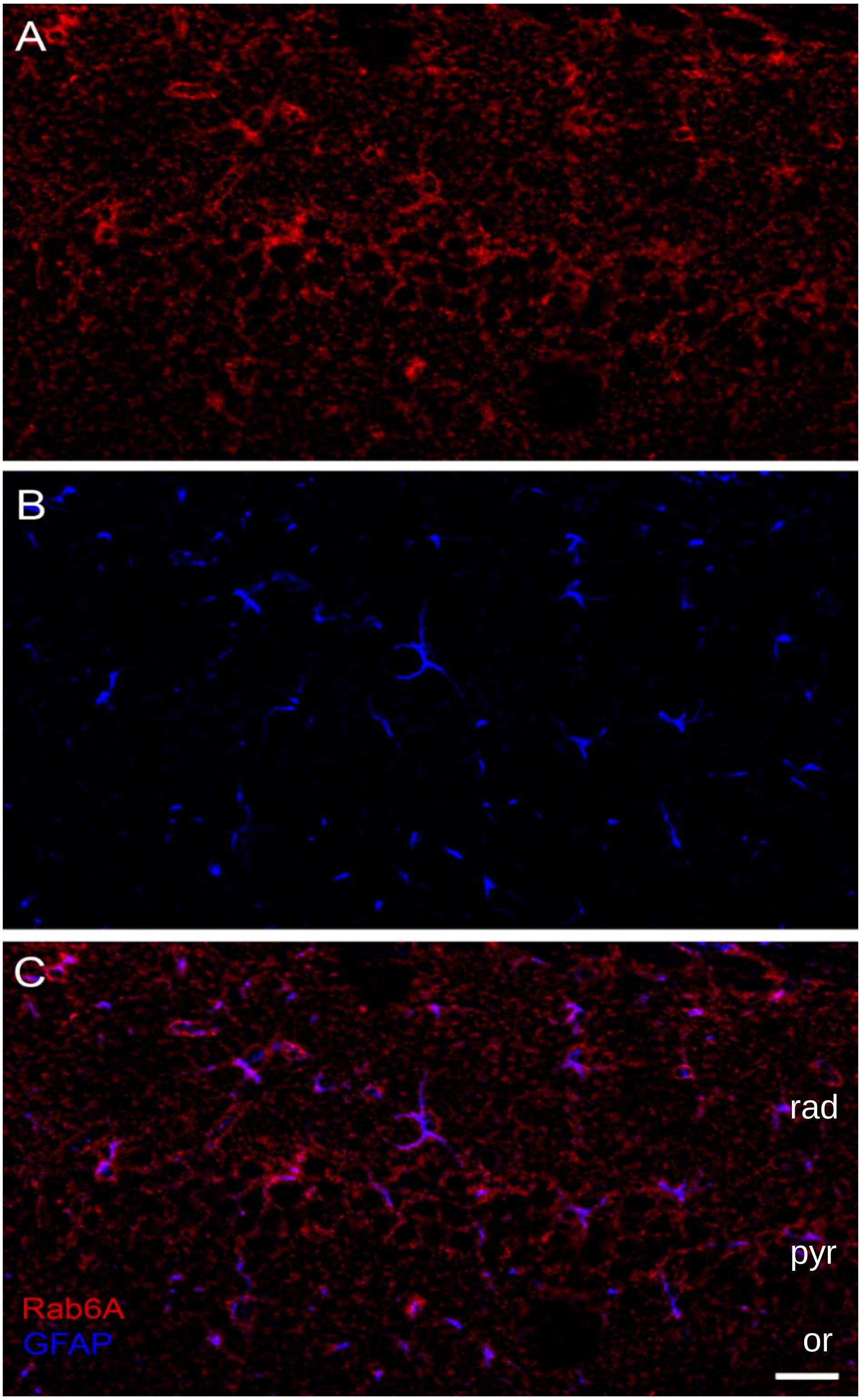

Figure S5. Rab6A localizes to GFAP+ astrocytes. At low magnification Rab6A+ structures (**a**) are hardly recognized as astrocytes. Astrocytic Rab6A staining is obvious only when overlaid with the GFAP channel (**b**), although many Rab6A+ grains are not double stained. Note the engulfing of pyramidal cells by Rab6A+ structures (**c**). Hippocampus, CA1; or, stratum oriens; pyr, stratum pyramidale; rad, stratum radiatum. Scale: 50 µm.

Fig. S5

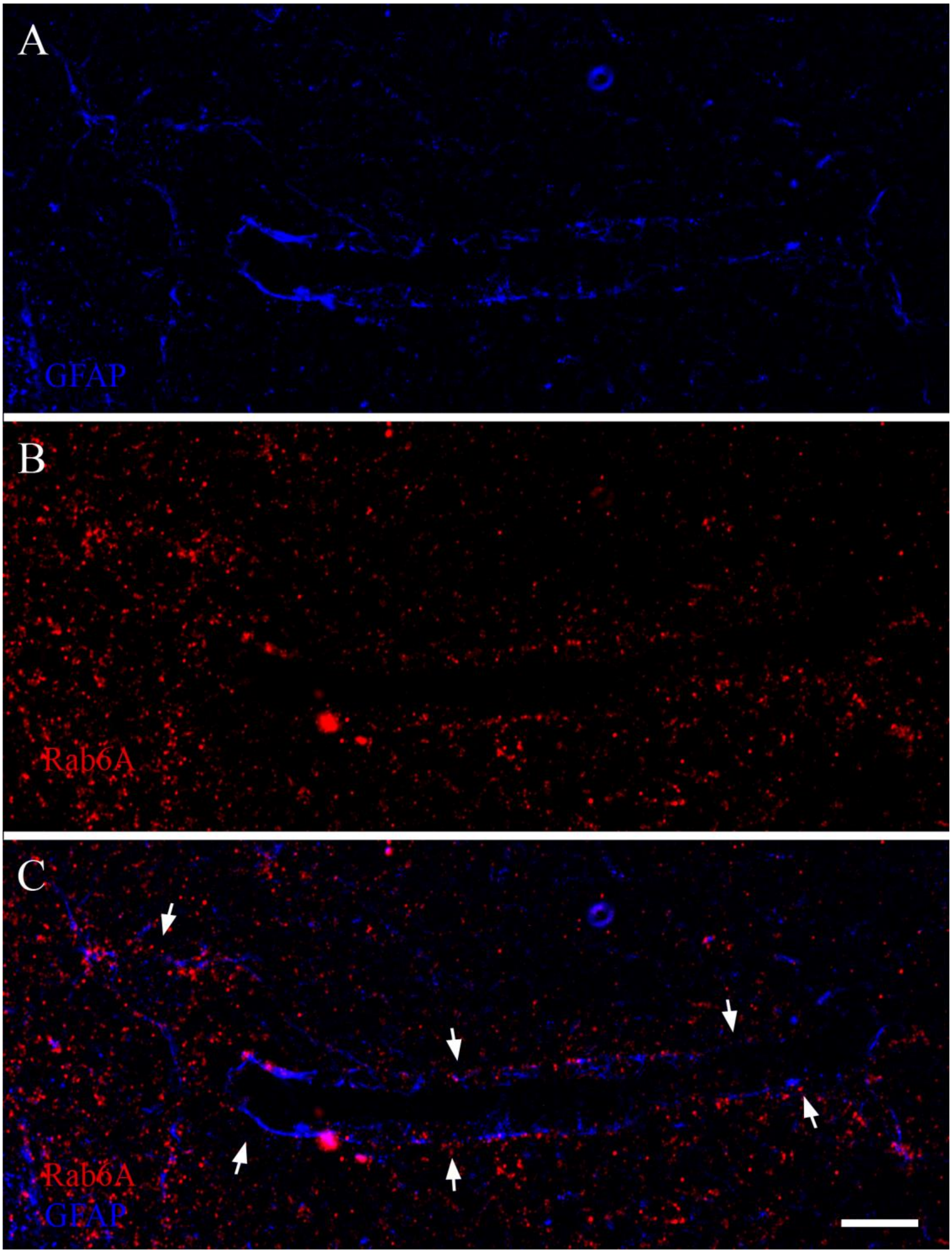

Figure S6. Rab6A+ in perivascular glial endfeet. GFAP+ glial endfeet abut a vessel wall (**a**). Rab6A+ puncta delineate the vessel (**b**) and colocalize with GFAP (arrows in **c**). Scale: 10 μm.

Fig. S6

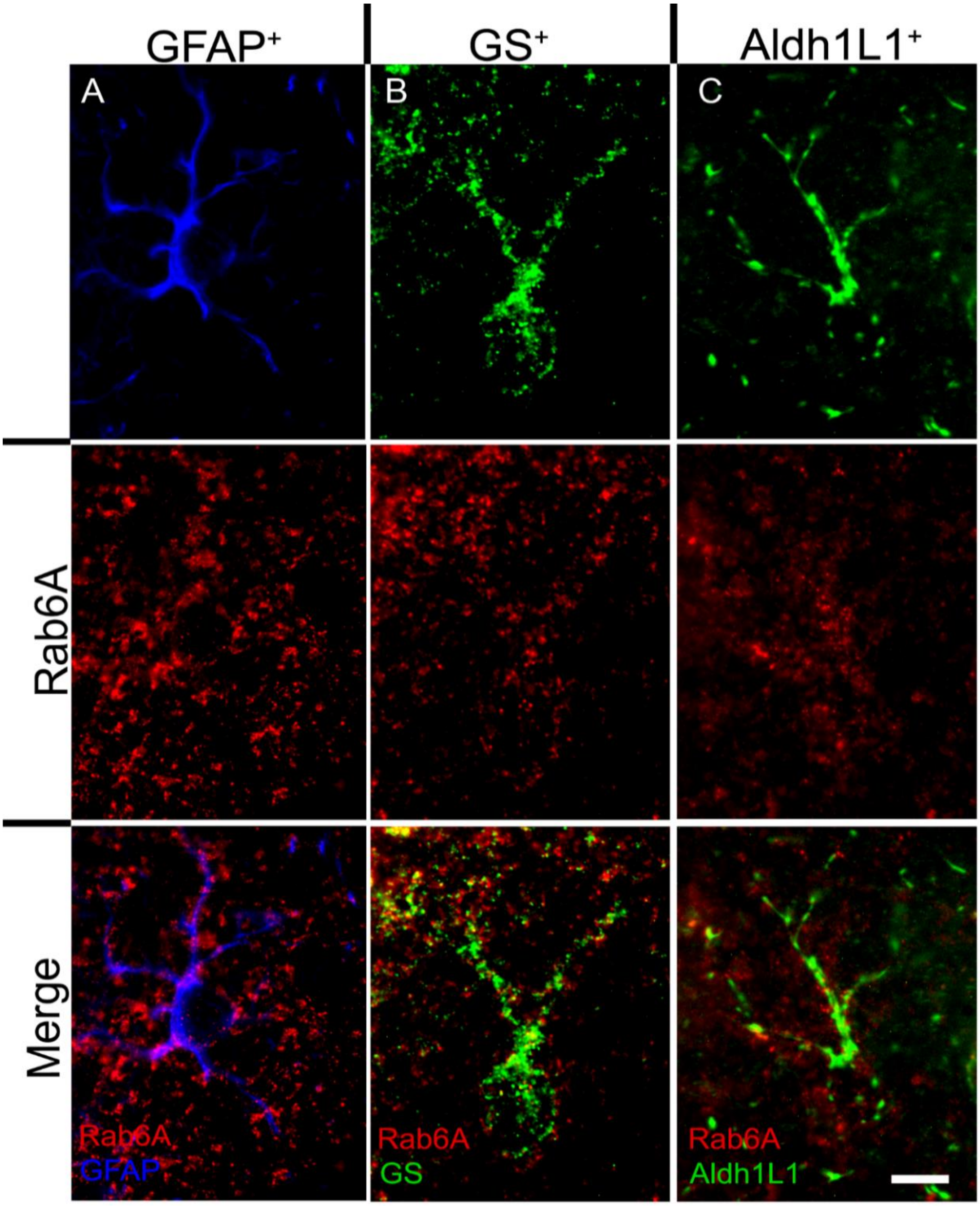

Figure S7. Rab6A localizes specifically to astrocytes. All astrocytes positive for GFAP (**a**), GS (**b**), or Aldh1L1 (**c**) display double label for Rab6A (see Diagrams S5, S6 for quantification).
Scale: 10 μm (in c, for a-c).

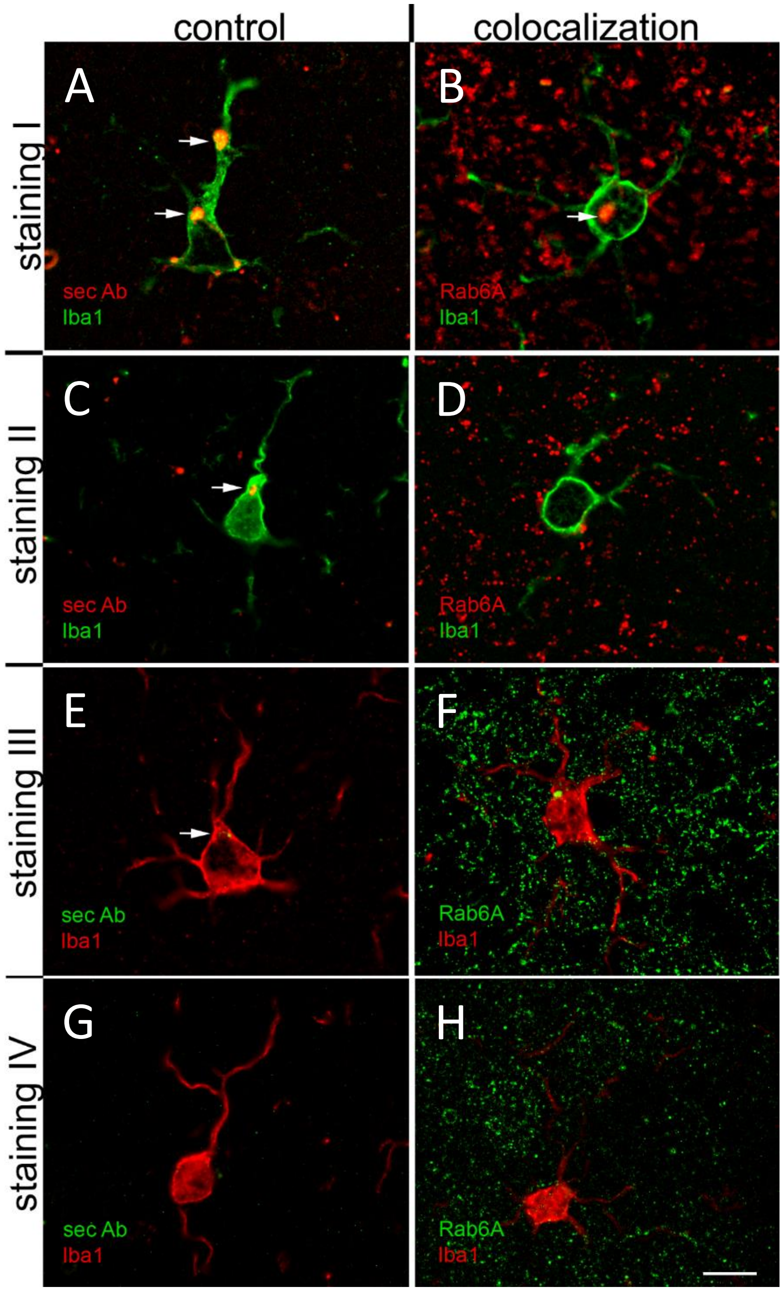

Figure S8. False positive Rab6A staining in microglia may be induced by the detection system. (**a - f**) In stainings I, II, and III, different sets of secondary antibodies were applied as specified in Table (below). A varying proportion of microglial cells contained Rab6A+ puncta (**b, d, f; see Table**). However, they are considered as selective, non-specific staining in microglia since they are also present in the parallel controls omitting anti-Rab6A (**a, c, e**). In addition, these non-specific puncta are rare within the individual microglial cell and frequently larger than the astrocytic Rab6A+ TGN structures in the surrounding neuropil (**a, b, c, f**). (**g, h**) Only with the detection system of staining IV could the selective, non-specific microglial staining be avoided (**see Table**). Scale: 10 µm.

Table Double immunostainings with false positive results in microglia

	pair of primary Abs	pair of corresponding secondary Abs	% false positive of all microglia (n) checked
staining I	**mouse-anti-Rab6A**<br>goat-anti-Iba1	**donkey-anti-mouse-Cy3**<br>donkey-anti-goat-Dy 488	65 % (58)
staining II		**horse-anti-mouse biotin + streptavidin-Cy3**<br>donkey-anti-goat-Dy 488	22 % (166)
staining III		**donkey-anti-mouse-Alexa 488**<br>horse-anti-goat-biotin + streptavidin-Cy3	5 % (219)
staining IV		***donkey-anti-mouse-Alexa 647***<br>*horse-anti-goat-biotin + streptavidin-Cy3*	*0 % (181)*

Fig. S8

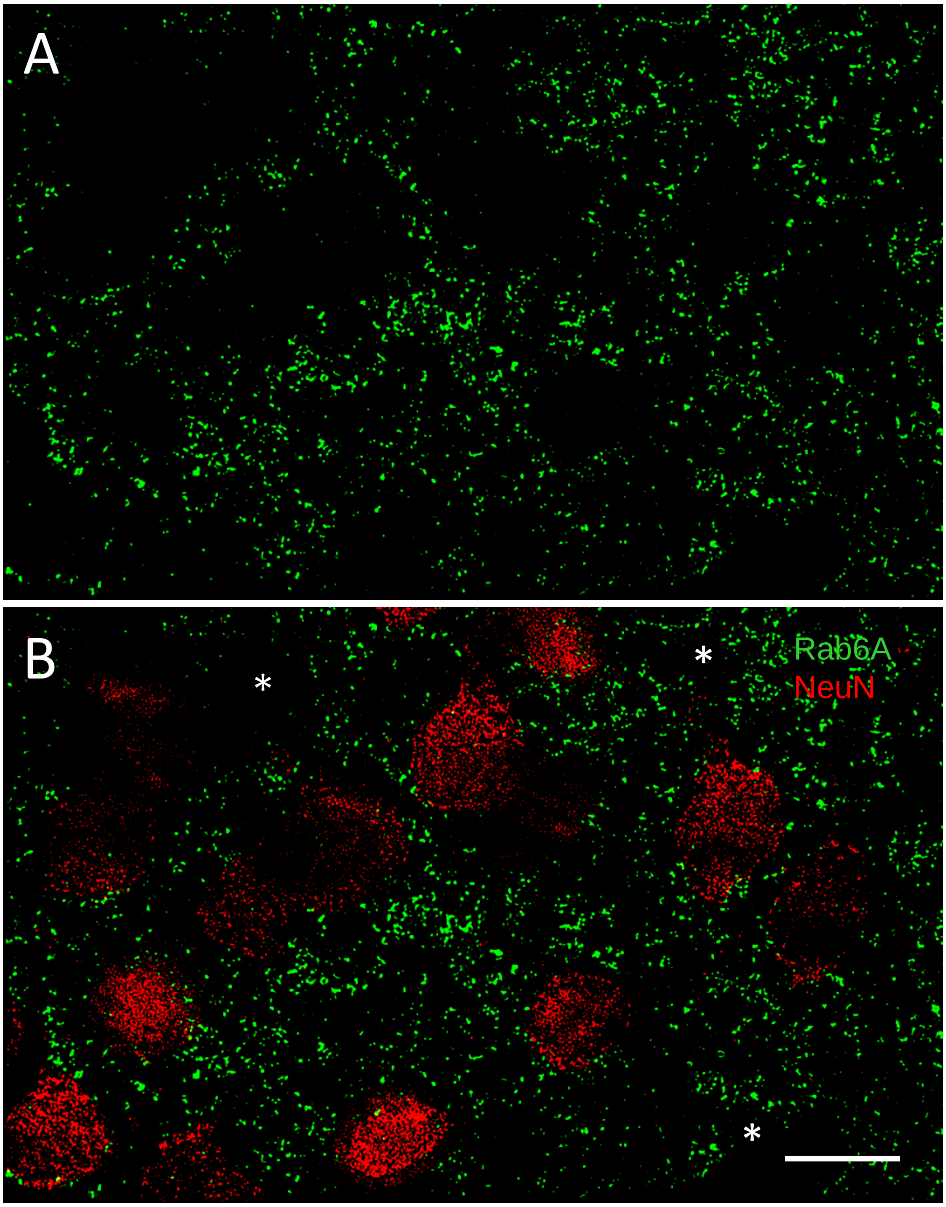

Figure S9. Neurons are Rab6-. (**a**) The ubiquitous Rab6A staining (green channel) displays many cut out holes. (**b**) They correspond to neuronal somata of pyramidal cells and interneurons visualized by NeuN (red channel), and to other cells or neuronal dendrites without label (some indicated by asterisks in **a**). Isocortex, high-resolution widefield microscopy and deconvolution. Scale: 10 µm.

Fig. S9

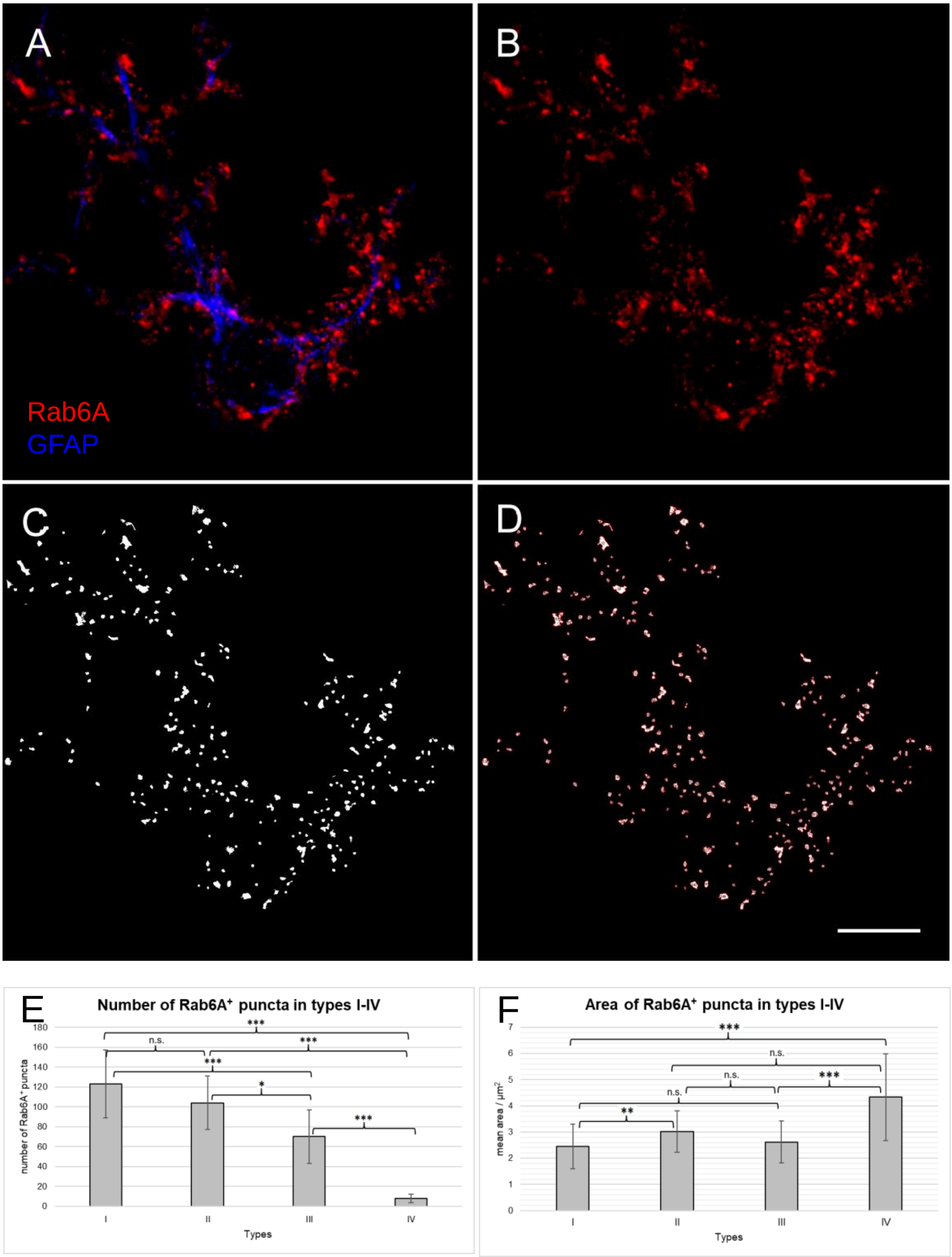

Figure S10. Quantitation of Rab6A$^+$ structures. Rab6A$^+$/GFAP$^+$ astrocytes with readily recognizable soma and processes were photodocumented applying identical microscopy and exposure settings, and isolated as ROIs (**a**). Analysis was done in the Rab6A channel (**b**), which was binarized based on the `local intensity maxima` (**c**). Objects thus obtained (red outlines, **d**) were counted and planimetrically evaluated. Image analysis was carried out using ImageJ (Rasband, 1997-2018) and identical parameters for all cells. Scale (in d, for a-d): 10µm.

Mean counts (**e**) and mean area (**f**) of Rab6A$^+$ puncta per ROI. For each of the four astrocyte types I – IV, 10 cells were analyzed. (**e**) The mean number of Rab6A$^+$ puncta is significantly different between all astrocyte types, except for I and II. Unifactorial ANOVA. (**f**) Area of Rab6A$^+$ puncta is significantly increased in type IV vs. types I and III, and in type II vs. type I. Kruskal-Wallis test, correction acc. to Bonferroni-Holm. (*) p<0,05, (**) p<0,01 und (***) p<0,001. n.s. non significant, with n=10.

Fig. S10

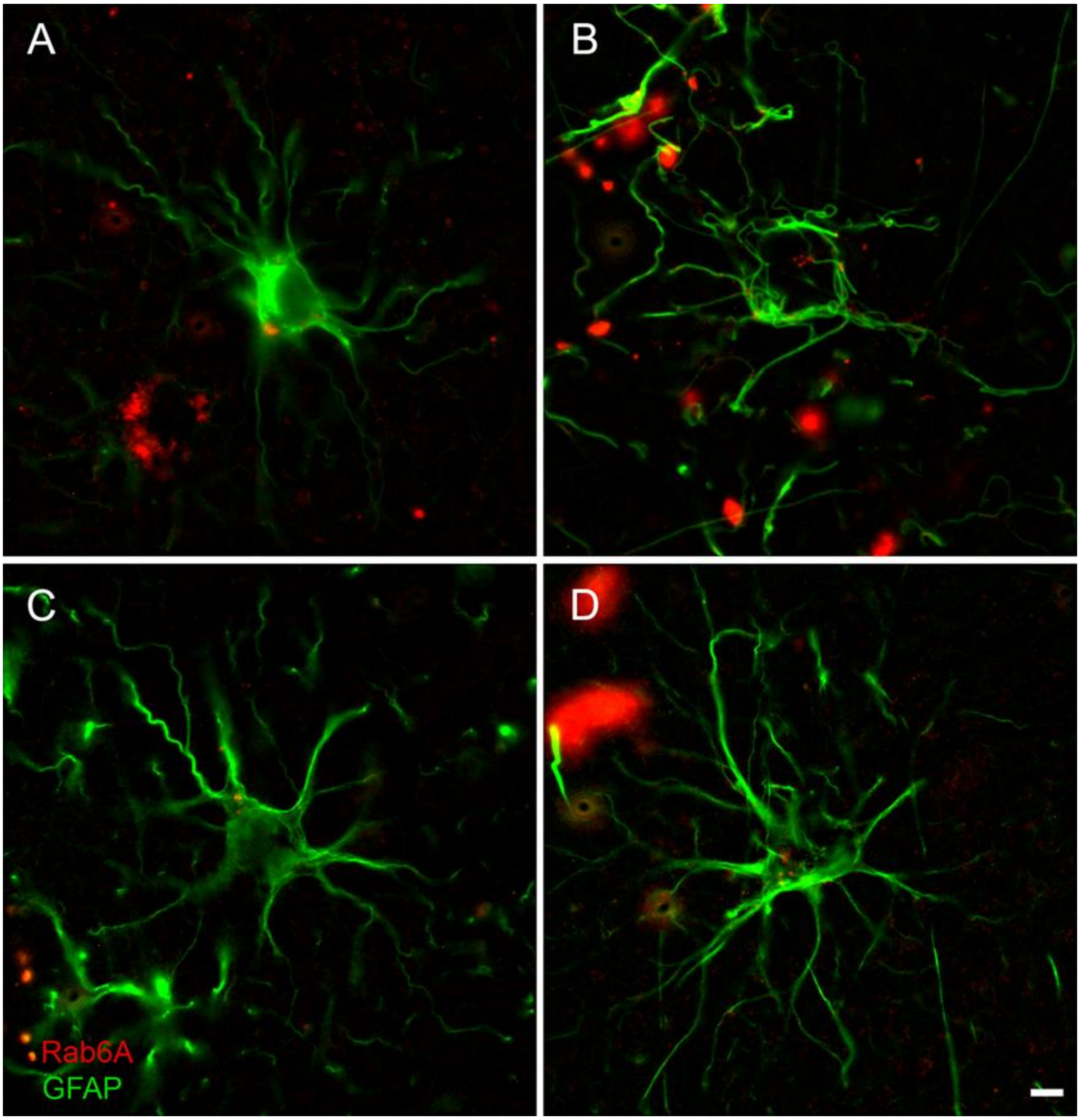

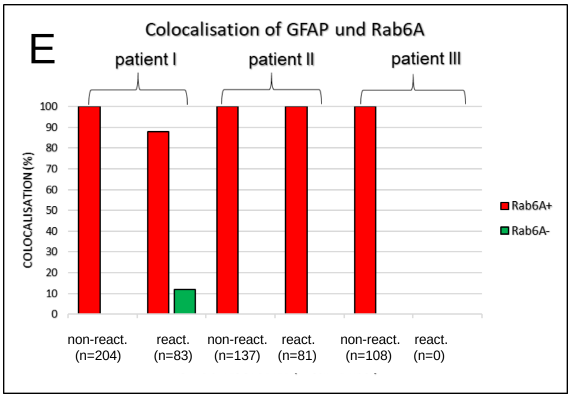

Figure S11. Some of the human GFAP+ astrocytes are Rab6A-. (**a-d**) Examples of the Rab6A- reactive GFAP+ astrocytes found only in case I. (**e**) Blinded quantification of Rab6A staining in preselected GFAP+ astrocytes. All other non-reactive or reactive astrocytes in cases II and III were Rab6A+, there were no reactive astrocytes in case III.
Scale: 10µm (d, for a-d).

Fig. S11

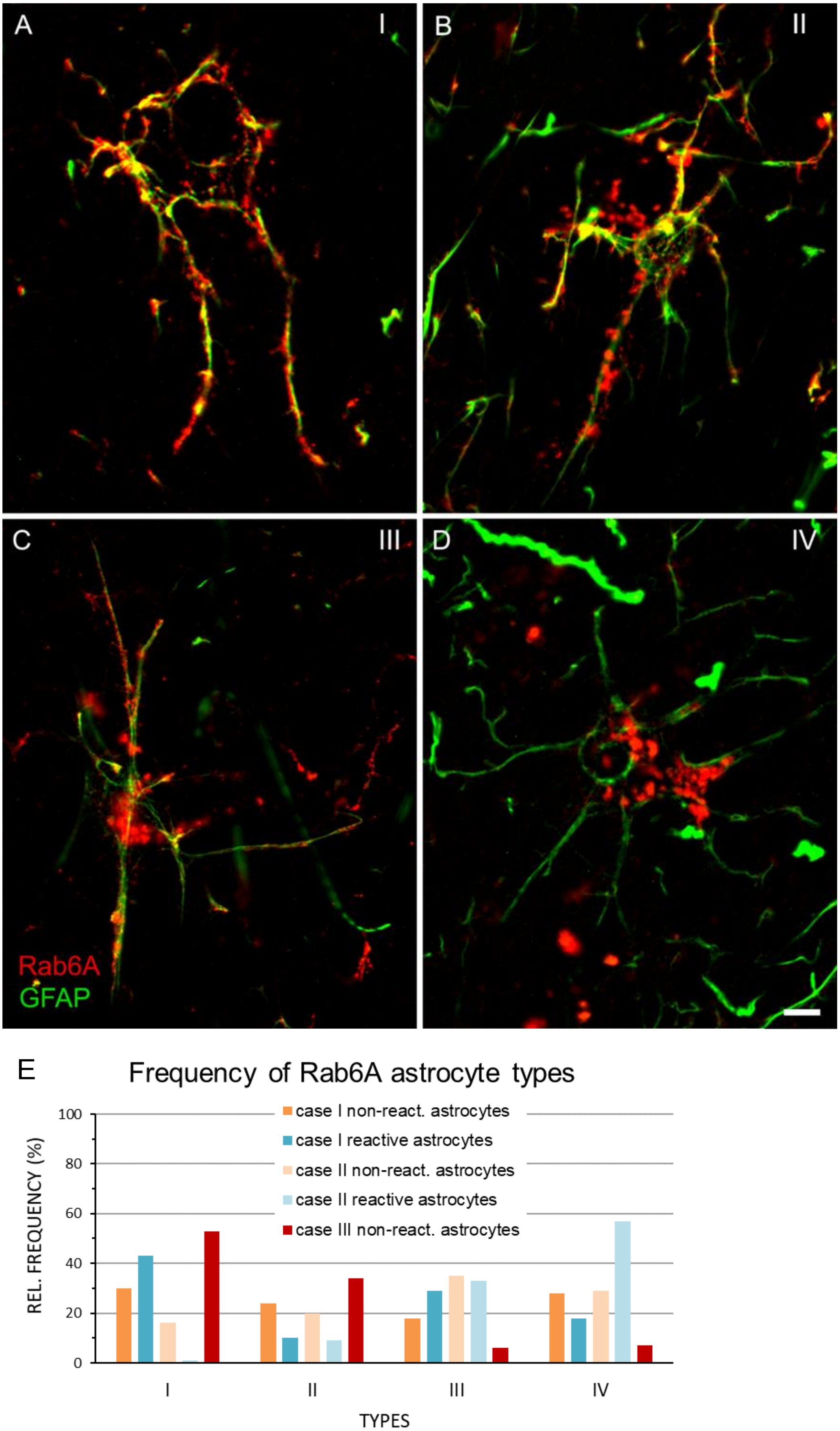

Figure S12. Classification of Rab6A⁺ astrocytes in human cortex. Rab6A⁺ astrocytes types I (**a**), II (**b**), III (**c**), and IV (**d**) (comp. Table 4) can also be discerned in human cortex, where their relative frequency differs between cases I-III (**e**). The decrease of frequency over types I-IV found in the mouse is reflected only in case III, which is without reactive astrocytes (see text). Scale: 5 µm (d, for a-d).

Fig. S12

Supplementary Materials: Table S1

Table S1 – Number of astrocytes (A) and non-astrocytic cells (B) checked

	A					B		
	GFAP$^+$	GS$^+$	Aldh1L1$^+$	Sox9$^+$		Iba1$^+$	NG2$^+$	CNPase$^+$
cell number	3742	3536	1116	6174		1136	299	1385
number of brain sections (double hemisphere)	12	6	6	6		8	4	6
animal number	3	3	3	3		5	2	5

Supplementary Materials: Diagrams S1 – S8

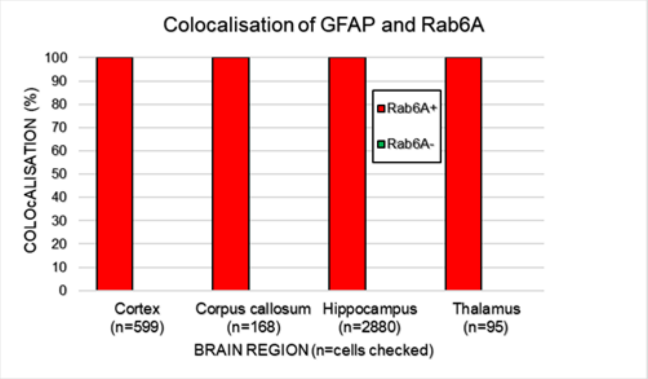

Diagram S1 – Localization of Rab6A in GFAP$^+$ astrocytes. All GFAP$^+$ astrocytes were Rab6A$^+$; there was not a single GFAP$^+$/Rab6A$^-$ astrocyte. GFAP$^+$ astrocytes were checked in various brain regions, in 12 tissue sections from 3 mice (n=number of cells).

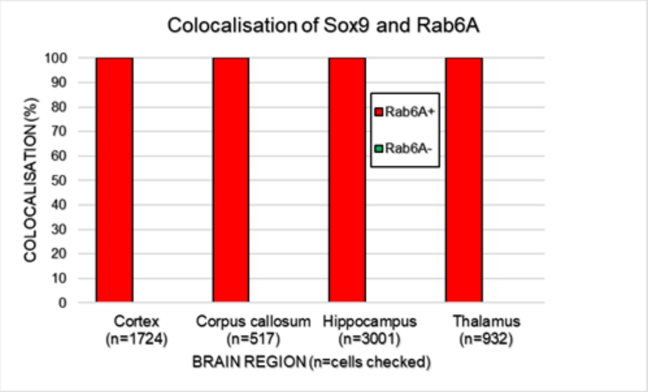

Diagram S2 - Localisation of Rab6A in Sox9$^+$ astrocytes. All SOX9$^+$ astrocytes were Rab6A$^+$; there

was not a single SOX$^+$/Rab6A$^-$ astrocyte. SOX9$^+$ astrocytes were checked in various brain regions, in 6 tissue sections from 3 mice (n=number of cells).

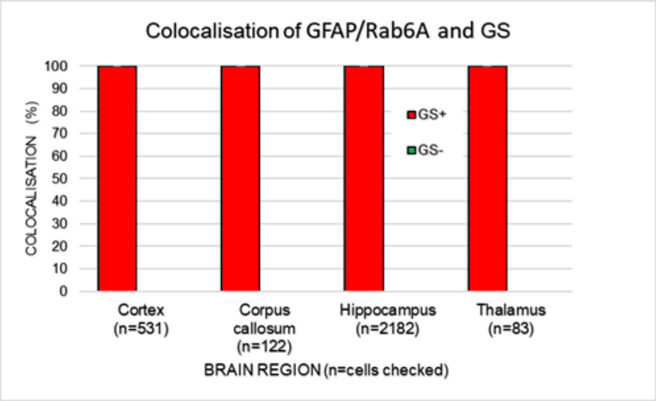

Diagram S3 - Localisation of GS in GFAP$^+$/Rab6A$^+$ astrocytes. All GFAP$^+$/Rab6A$^+$ astrocytes were GS$^+$; there was not a single GFAP$^+$/Rab6A$^+$/GS$^-$ astrocyte. GFAP$^+$/Rab6A$^+$ astrocytes were checked in various brain regions, in 6 tissue sections from 3 mice (n=number of cells).

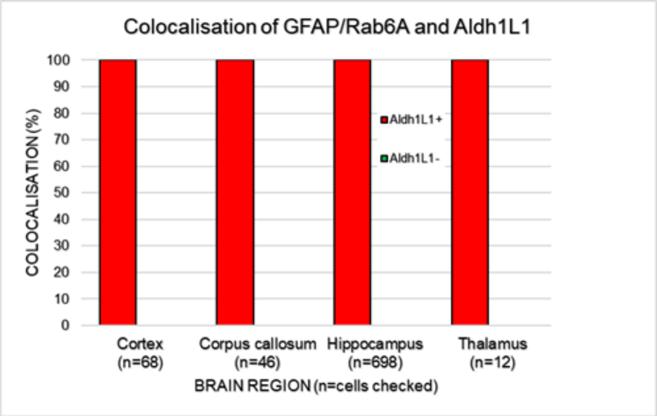

Diagram S4 - Localisation of Aldh1L1 in GFAP$^+$/Rab6A$^+$ astrocytes. All GFAP$^+$/Rab6A$^+$ astrocytes were Aldh1L1$^+$; there was not a single GFAP$^+$/Rab6A$^+$/ Aldh1L1$^-$ astrocyte. GFAP$^+$/Rab6A$^+$ astrocytes were checked in various brain regions, in 6 tissue sections from 3 mice (n=number of cells).

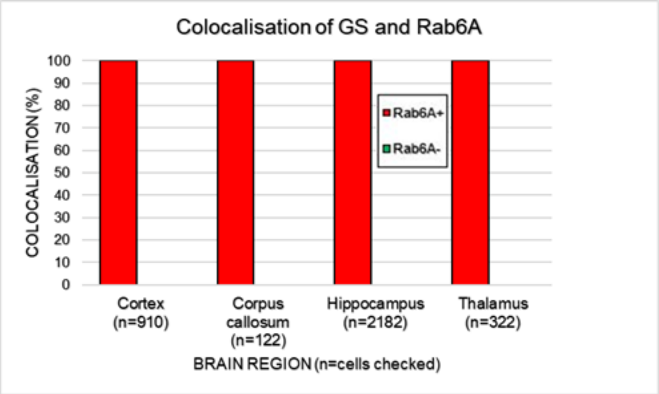

Diagram S5 - Localisation of Rab6A in GS$^+$ astrocytes. All GS$^+$ astrocytes were Rab6A$^+$; there was not a single GS$^+$/Rab6A$^-$ astrocyte. GS$^+$ astrocytes were checked in various brain regions, in 6 tissue sections from 3 mice (n=number of cells).

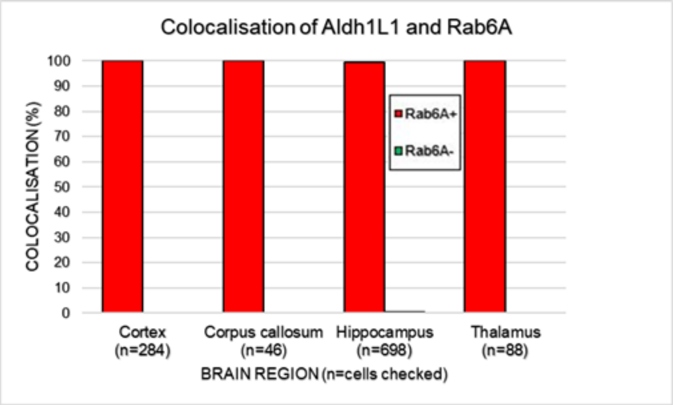

Diagram S6 - Localisation of Rab6A in Aldh1L1$^+$ astrocytes. All Aldh1L1$^+$ astrocytes were Rab6A$^+$; there was not a single Aldh1L1$^+$/Rab6A$^-$ astrocyte. Aldh1L1$^+$ astrocytes were checked in various brain regions, in 6 tissue sections from 3 mice (n=number of cells).

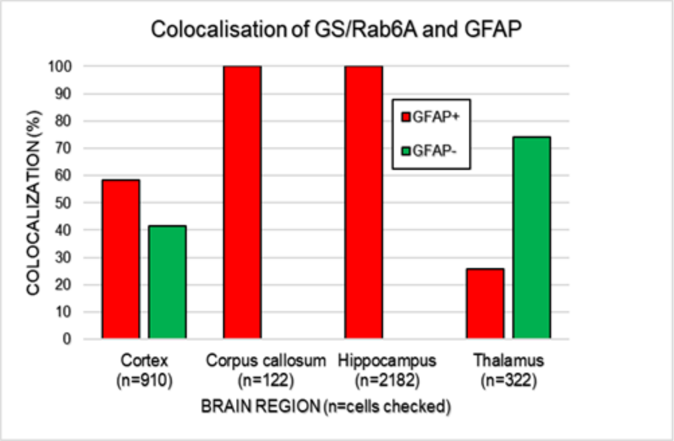

Diagram S7 - Localisation of GFAP in GS$^+$/Rab6A$^+$ astrocytes. In cortex and thalamus, not all GS$^+$/Rab6A$^+$ astrocytes display GFAP$^+$ label. All GS$^+$/Rab6A$^+$ astrocytes display GFAP$^+$ label in hippocampus and corpus callosum. GS$^+$/Rab6A$^+$ astrocytes were checked in various brain regions, in 6 tissue sections from 3 mice (n=number of cells).

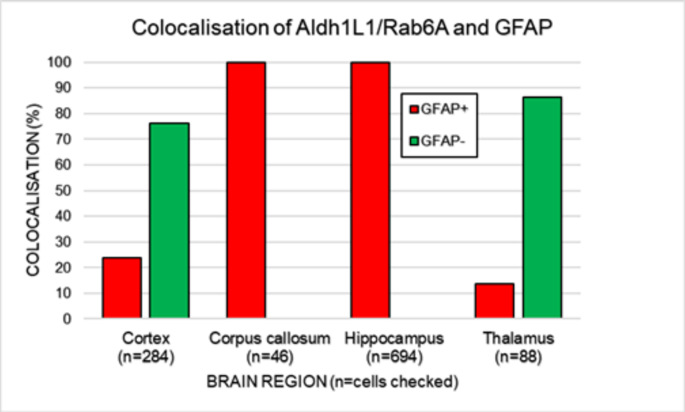

Diagram S8 - Localisation of GFAP in Aldh1L1$^+$/Rab6A$^+$ astrocytes. In cortex and thalamus, not all Aldh1L1$^+$/Rab6A$^+$ astrocytes display GFAP$^+$ label. All Aldh1L1$^+$/Rab6A$^+$ astrocytes display GFAP$^+$ label in hippocampus and corpus callosum. Aldh1L1/Rab6A$^+$ astrocytes were checked in various brain regions, in 6 tissue sections from 3 mice (n=number of cells).
